# Increased referrals for congenital hyperinsulinism genetic testing in children with trisomy 21 reflects the high burden of non‐genetic risk factors in this group

**DOI:** 10.1111/pedi.13333

**Published:** 2022-03-23

**Authors:** Thomas I. Hewat, Thomas W. Laver, Jayne A. L. Houghton, Jonna M. E. Männistö, Sabah Alvi, Stephen P. Brearey, Declan Cody, Antonia Dastamani, Miguel De los Santos La Torre, Nuala Murphy, Birgit Rami‐Merhar, Birgit Wefers, Hanna Huopio, Indraneel Banerjee, Matthew B. Johnson, Sarah E. Flanagan

**Affiliations:** ^1^ Institute of Biomedical and Clinical Science University of Exeter Medical School Exeter UK; ^2^ Exeter Genomics Laboratory Royal Devon and Exeter NHS Foundation Trust Exeter UK; ^3^ Department of Pediatrics University of Eastern Finland and Kuopio University Hospital Kuopio Finland; ^4^ Leeds Children's Hospital Leeds UK; ^5^ Countess of Chester Hospital Chester UK; ^6^ Children's Health Ireland at Crumlin Dublin Ireland; ^7^ Endocrinology Department Great Ormond Street Hospital for Children NHS Foundation Trust London UK; ^8^ Department of Endocrinology and Metabolism of the Instituto Nacional de Salud del Niño Lima Peru; ^9^ Children's University Hospital Dublin Ireland; ^10^ Department of Pediatric and Adolescent Medicine Medical University of Vienna Vienna Austria; ^11^ Ninewells Hospital Dundee UK; ^12^ Department of Pediatrics Kuopio University Hospital Kuopio Finland; ^13^ Department of Paediatric Endocrinology Royal Manchester Children's Hospital Manchester UK

## Abstract

**Background:**

Hyperinsulinism results from inappropriate insulin secretion during hypoglycaemia. Down syndrome is causally linked to a number of endocrine disorders including Type 1 diabetes and neonatal diabetes. We noted a high number of individuals with Down syndrome referred for hyperinsulinism genetic testing, and therefore aimed to investigate whether the prevalence of Down syndrome was increased in our hyperinsulinism cohort compared to the population.

**Methods:**

We identified individuals with Down syndrome referred for hyperinsulinism genetic testing to the Exeter Genomics Laboratory between 2008 and 2020. We sequenced the known hyperinsulinism genes in all individuals and investigated their clinical features.

**Results:**

We identified 11 individuals with Down syndrome in a cohort of 2011 patients referred for genetic testing for hyperinsulinism. This represents an increased prevalence compared to the population (2.5/2011 expected vs. 11/2011 observed, *p* = 6.8 × 10^−5^). A pathogenic *ABCC8* mutation was identified in one of the 11 individuals. Of the remaining 10 individuals, five had non‐genetic risk factors for hyperinsulinism resulting from the Down syndrome phenotype: intrauterine growth restriction, prematurity, gastric/oesophageal surgery, and asparaginase treatment for leukaemia. For five individuals no risk factors for hypoglycaemia were reported although two of these individuals had transient hyperinsulinism and one was lost to follow‐up.

**Conclusions:**

Down syndrome is more common in patients with hyperinsulinism than in the population. This is likely due to an increased burden of non‐genetic risk factors resulting from the Down syndrome phenotype. Down syndrome should not preclude genetic testing as coincidental monogenic hyperinsulinism and Down syndrome is possible.

## 1 INTRODUCTION

Hyperinsulinism (HI) is a disorder of the pancreatic beta‐cell where inappropriately high levels of insulin are secreted leading to hypoglycaemia. Prolonged neonatal HI can be transient, often remitting within 6 months, with risk factors including male sex, low birth weight, and perinatal stress.^1^ In contrast, persistent HI is likely to be genetic with disease‐causing mutations in single genes identified in 50%–70% of cases.^2^, ^3^ HI has also been reported as a rare feature in patients with aneuploidies. For example, HI can present in females with Turner syndrome resulting from a complete or partial monosomy of the X chromosome and in children with Patau syndrome resulting from mosaic trisomy 13.^4^, ^5^

The most common aneuploidy is trisomy 21, causing Down syndrome, which affects 1 in 794 live births in the USA.^6^ Down syndrome is characterized by intellectual disability, microcephaly, congenital heart defects, gastrointestinal disorders, and endocrine disorders which include Type 1 diabetes or neonatal diabetes.^7^, ^8^, ^9^ Whilst HI has not been reported as a feature of Down syndrome, we noted a high number of individuals with the co‐existence of these two conditions being referred to our laboratory for genetic testing. Our aim was to assess whether the prevalence of children with HI and Down syndrome was higher than expected in our cohort and if so to determine the reason(s) for this.

## 2 METHODS

We studied 2011 individuals referred for HI genetic testing to the Exeter Genomics Laboratory between 2008 and 2020. Clinical information was provided at referral using a standardized request form. Follow‐up data by case note review were requested for all individuals with HI and Down syndrome.

We performed targeted next‐generation sequencing of 13 known HI genes including *ABCC8*, *CACNA1D*, *CDKN1C*, *GCK*, *GLUD1*, *HADH*, *HNF1A*, *HNF4A*, *INSR*, *KCNJ11*, *PMM2*, *SLC16A1*, and *TRMT10A* in all individuals with HI and Down syndrome using previously described methods.^10^ We used Stata/SE v16.0 to perform a one‐sample binomial test to assess if the prevalence of Down syndrome in our cohort was significantly higher than the population prevalence (Stata Corp, College Station, TX, USA).

Informed consent was obtained from the parents or guardians of all probands. This study was approved by the North Wales Research Ethics Committee (517/WA/0327).

## 3 RESULTS

Within our international cohort of 2011 individuals, we identified 11 cases with Down syndrome (*n* = 11/2011 [0.55%]). This represents a minimal prevalence as we do not routinely screen for aneuploidies, and some clinicians may not have provided this information on the genetic request form. The number of children with Down syndrome was significantly higher than expected by chance given the population prevalence of Down syndrome of 12.6/10,000^6^ (2.5/2011 expected vs. 11/2011 observed, *p* = 6.8 × 10^−5^).

We identified a mutation in a known HI gene in 1/11 (9%) patients. This individual had a pathogenic paternally inherited *ABCC8* mutation.^11^ Of the 10 individuals without a mutation in a known gene, two were born with intrauterine growth retardation (IUGR) (birth weight *Z*‐score < −2). The median age at diagnosis at HI of the 10 individuals was 101 days (IQR 1–581 days) with insulin detected at the time of hypoglycaemia (plasma glucose <2.8 mmol/L) in all cases. Persistent HI (defined here as requiring treatment for >6 months) was confirmed in four of the 10 genetically unsolved individuals. In the remaining six individuals the HI was transient (*n* = 5) or follow‐up information was not available (*n* = 1). One individual with persistent HI demonstrated side‐effects to diazoxide and did not respond to octreotide, necessitating a near‐total pancreatectomy.^12^ Consanguinity was reported in this individual.

Seven individuals, including the child with an *ABCC8* mutation, had undergone gastric or oesophageal surgery for duodenal atresia, duodenal stenosis, tracheomalacia, or gastro‐oesophageal reflux disease (GORD). In two cases surgery had been performed prior to the onset of HI. One of these cases had also undergone surgery to repair a portosystemic shunt.^13^ A further individual had been diagnosed with acute lymphoblastic leukaemia and had received L‐asparaginase treatment prior to the onset of HI. An overview of the clinical features of the cohort are provided in Table 1.

**TABLE 1:** Clinical features of patients diagnosed with Down syndrome and Hyperinsulinism

	Patient 1	Patient 2	Patient 3	Patient 4	Patient 5	Patient 6	Patient 7	Patient 8	Patient 9	Patient 10	Patient 11
Genetic results	No mutation detected	No mutation detected	No mutation detected	No mutation detected	No mutation detected	No mutation detected	No mutation detected	No mutation detected	No mutation detected	No mutation detected	*ABCC8* p.G25fs/N**
Sex	Female	Female	Male	Male	Female	Female	Male	Female	Male	Female	Male
Birth weight SDS	−2.55	Not available	1.35	0.25	−0.77	0.22	−1.07	0.89	−3.74	1.25	0.65
Age at HI diagnosis (weeks)	36	20	120	0.14	83	0.14	0.14	0.14	9	208	0.43
Glucose (mmol/L) (Insulin [pmol/L]) at diagnosis	2 (108)	1.5 (60.5)	1 (18.3)	0.4 (26)	1.6 (88)	1.9 (12.8)	2.0 (347)	1.8 (12.5)	2.8 (4.5)	2.4 (141)	1.1 (56.1)
Post‐prandial hypoglycaemia	Not noted	Not noted	No	No	Yes	Not noted	Not noted	Not noted	Not noted	Not noted	Not noted
Transient/persistent HI	**Persistent** Diazoxide treatment ongoing at 13 years (3 mg/kg/day)	**Transient** No treatment required	**Persistent** Pancreatectomy due to side effects of diazoxide and no response to octreotide	**Transient** Treated transiently with I.V. glucose and increased feeds	**Persistent** Diazoxide unresponsive, managed with continuous feeds until remission at 3 years following VSD correction	**Transient** Treated with diazoxide until 4 months	**Transient** No treatment required	**Unknown** Lost to follow‐up (10 mg/kg/day diazoxide at referral)	**Persistent** Treated with diazoxide, until 12 months	**Transient** Treated transiently with I.V. glucose	**Persistent** Octreotide (20ug/kg/day) ongoing at 5 years
Gastric or oesophageal surgery (age)	No	Yes, prior to HI diagnosis (16 weeks)	Yes, following HI diagnosis (3 years)	No	Yes, prior to HI diagnosis (1st, 4 weeks, 2nd 1 year)	Yes, following HI diagnosis (>0.14 weeks)	No	Yes (age unknown)	Yes, following HI diagnosis (26 weeks)	No	Yes, following HI diagnosis (0.86 weeks)
Additional features	ASD	GORD, VSD	West syndrome, GORD, asthma	Mild hypoventilation, ASD	Tracheo‐oesophageal fistula, GORD, VSD, jaundice, portosystemic shunt	Duodenal atresia, ASD, PDA	Prematurity (31/40), perinatal compromise (poor CTG, reduced movements, at birth: raised lactate, biochemical evidence of liver & renal compromise) Cerebral palsy with right hemiplegia 2nd to left Periventricular Leukomalacia, Cataracts, Hearing loss 2nd auditory neuropathy	Duodenal stenosis, haematuria	Tracheomalacia	Acute lymphoblastic leukaemia treated with L‐asparaginase at 3.8 years	Duodenal atresia

*Note*: Gray‐filled boxes represent risk factors for Hyperinsulinism (HI). ASD = atrial septal defect. VSD = ventricular septal defect. PDA = patent ductus arteriosus. GORD = gastro‐oesophageal reflux disease. I.V. intravenous. For the purposes of this study persistent disease is defined as HI requiring treatment for >6 months and transient disease is defined as HI requiring treatment for <6 months. Patient 3 previously reported in.^12^ Patient 5 previously reported in.^13^ ** indicates that mutation was previously reported in.^11^

## 4 DISCUSSION

We identified 11 individuals with HI and Down syndrome. Given that Down syndrome has an approximate incidence of one in 794 live births, we would have expected two or three individuals with Down syndrome in our cohort of 2011 individuals.^6^ The statistically significant enrichment and higher prevalence therefore suggest that the two conditions are related.

The prevalence of mutations in the known genes was low in the Down syndrome and HI cohort (*n* = 1/11, 9%) although this increased to 20% in those with confirmed persistent HI (*n* = 1/5). This pick‐up rate is lower than anticipated given previous studies have reported mutations in the known genes in 50%–70% of HI cases.^2^, ^3^ While this may reflect the small sample size, it is also possible that the Down syndrome is increasing the risk of the child developing HI.

We identified risk factors for developing HI in five of the 10 individuals without a mutation in a known gene. Two children had surgery to correct a gastrointestinal (GI) disorder prior to the onset of HI (Table 1). GI disorders are common in individuals with Down syndrome and surgical management of this can lead to iatrogenic hypoglycaemia as a result of dumping syndrome.^14^, ^15^ Furthermore, one of these individuals had confirmed post‐prandial hypoglycaemia following surgery lending further support to this diagnosis.^16^ This patient also had a portosystemic shunt, with surgical closure resulting in a resolution of the hypoglycaemia.^13^ In four further cases, gastric surgery was performed but this occurred after the onset of HI in three cases suggesting that the HI was unlikely to be due to gastric surgery induced post‐prandial hypoglycaemia. The age at gastric surgery in the remaining patient was unknown.

IUGR or biochemical evidence of perinatal and postnatal stress associated with prematurity, was reported in two individuals. These are well‐recognized risk factors for prolonged neonatal hypoglycaemia.^1^ IUGR was reported in a second individual however the HI was ongoing at the age of 13 years suggesting it was not causative of the hypoglycaemia.^17^

One individual had been diagnosed with acute lymphoblastic leukaemia that had been treated with an L‐asparaginase based chemotherapy prior to the onset of HI at 4 years. Children with Down syndrome are at increased risk of developing acute lymphoblastic leukaemia and previous studies have shown that treatment with L‐asparaginase can cause hypoglycaemia in younger patients.^18^, ^19^ This could explain the transitory hypoglycaemia observed in this child.

Of the five individuals without an identifiable risk factor for HI, two had persistent HI, two had transient HI and one case was lost to follow‐up which might suggest that the HI was transient and not severe. It is also possible that in this patient risk factors for HI were present but not reported at referral for genetic testing. The finding of two individuals with Down syndrome and persistent HI within our cohort is expected based on the population prevalence of Down syndrome. Interestingly, consanguinity was reported in one of these individuals, supporting the possibility of a recessively inherited monogenic etiology.

Recently, a study of HI in Finland identified five cases with Down syndrome in a cohort of 238 individuals. The authors noted that this was a statistically significant increase compared to the population prevalence of Down syndrome.^20^ In keeping with our findings, screening of the known genes identified an *ABCC8* mutation in a single individual whilst the four mutation negative individuals had non‐genetic risk factors for HI which could be attributed to the Down syndrome phenotype: extreme prematurity and cardiac insufficiency, IUGR, gastric surgery/fundoplication, and stress due to congenital heart defects (personal communication Huopio and Männistö). In two individuals the HI remitted before the age of 4 months.

Genetic testing identified an *ABCC8* mutation in one individual with Down syndrome in our cohort and this, together with the finding of an *ABCC8* mutation in an individual within the Finnish cohort, highlights the need to perform genetic testing in all individuals with persistent HI.^20^ Whilst a diagnosis of Down syndrome does not preclude co‐incidental monogenic HI, our study suggests HI in Down syndrome is most likely to be due to non‐genetic risk factors.

In conclusion, we have identified an increased referral rate for HI genetic testing for individuals with Down syndrome. Our findings suggest that HI is not a feature of Trisomy 21 but a consequence of the high burden of non‐genetic risk factors resulting from the Down syndrome phenotype.

### FUNDING STATEMENT

TWL is the recipient of a Lectureship and MBJ an Independent Fellowship from the Exeter Diabetes Centre of Excellence funded by Research England's Expanding Excellence in England (E3) fund. SEF has a Sir Henry Dale Fellowship jointly funded by the Wellcome Trust and the Royal Society (Grant Number: 105636/Z/14/Z). This research was funded in whole, or in part, by Wellcome [105636/Z/14/Z]. For the purpose of open access, the author has applied a CC BY public copyright license to any Author accepted Manuscript version arising from this submission.

## CONFLICT OF INTEREST

The authors have nothing to disclose.

## AUTHOR CONTRIBUTION

T.I. Hewat, M.B. Johnson, and S.E. Flanagan designed the study. S. Alvi, S.P. Brearey, D. Cody, A. Dastamani, M. de los Santos la Torre, N. Murphy, B. Rami‐Merhar, B. Wefers, I. Banerjee and S.E. Flanagan recruited patients to the study and with J.M.E Männistö and H. Huopio analysed the clinical data. J.A.L. Houghton and S.E. Flanagan performed the molecular genetic studies. T.I. Hewat, T.W. Laver, M.B. Johnson and S.E. Flanagan performed the data analysis. T.I. Hewat, M.B. Johnson and S.E. Flanagan prepared the draft manuscript. All authors contributed to the discussion of the results and to the manuscript preparation.

## ETHICAL APPROVAL

This study was approved by the North Wales Research Ethics Committee (517/WA/0327).

## PATIENT CONSENT STATEMENT

Informed consent was obtained from the parents or guardians of all probands.

## Data Availability

The data that support the findings of this study are available from the corresponding author upon reasonable request.
